# Proteotyping to Establish Gene Origin within Reassortant Influenza Viruses

**DOI:** 10.1371/journal.pone.0015771

**Published:** 2011-01-31

**Authors:** Ji-won Ha, Alexander B. Schwahn, Kevin M. Downard

**Affiliations:** School of Molecular Bioscience, University of Sydney, Sydney, New South Wales, Australia; Erasmus Medical Center, The Netherlands

## Abstract

The application of a rapid and direct proteotyping approach with which to identify the gene origin of viral antigens in a reassortant influenza strain is demonstrated. The reassortant strain, constructed for a vaccine against type A 2009 H1N1 pandemic influenza, contains genes derived from a wild-type pandemic strain (A/California/7/2009) and an egg adapted high-growth strain (denoted NYMC X-157) derived from an earlier A/Puerto Rico/8/34 strain. The proteotyping approach employs modern proteomics methods and high resolution mass spectrometry to correctly establish that the genes of the surface antigens, hemagglutinin and neuraminidase, are derived from the A/California/7/2009 strain while those for nucleoprotein and matrix protein M1 antigens are derived from the NYMC X-157 strain. This is achieved for both gel-separated antigens and those from a whole vaccine digest. Furthermore, signature peptides detected in the mass spectra of the digested antigens enable the engineered reassortant strain to be identified as a type A virus of the H1N1 subtype in accord with earlier studies. The results demonstrate that proteotyping approach provides a more direct and rapid approach over RT-PCR with which to characterize reassortant strains of the influenza virus at the molecular protein level. Given that these strains pose the greatest risk to human and animal health and have been responsible for all human pandemics of the 20th and 21st centuries, there is a vital need for the origins and evolutionary history of these strains to be rapidly established.

## Introduction

In addition to antigenic drift [1], caused by errors in viral replication and the antigenic pressure applied to the surface hemagglutinin and neuraminidase antigens by the immune response, the evolution of the influenza virus is shaped by the reassortment process [2], [3]. The virus has a high potential to reassort due to the segmented nature of its genome that consists of eight negative-strand RNA segments. When two different strains of influenza virus co-infect the same cell, progeny viruses (reassortants) are created that contain genes derived from each parent. Genetic reassortment among influenza viruses occurs naturally and plays an important role in viral epidemiology and pathogenicity [4].

All of the pandemics of the 20th and 21st century have resulted from reassorted type A influenza viruses of the H1N1, H2N2, and H3N2 subtypes [5]. The most recent 2009 influenza pandemic strain originated around 1998 when the swine influenza viruses reassorted with a human type A (H3N2) influenza virus and an avian influenza virus of an unknown subtype. This produced a triple reassorted H3N2 virus in swine populations throughout North America [6]. Subsequent reassortment with a H1N1 swine strain of the virus resulted in the generation of the triple reassorted swine A (H1N1) that caused the 2009 H1N1 influenza pandemic [6].

There is increasing concern that the 2009 pandemic strain could reassort with seasonal strains to produce a deadly new strain of the virus. In the southern hemisphere, the pandemic strain dominated the influenza season providing an opportunity for reassortment with later seasonal strains [7]. Of particular concern is if the pandemic strain acquires the neuraminidase gene from seasonal influenza and establishes some resistance to anti-viral inhibitors [8]. The ability to rapidly characterize reassortant strains of the virus and assess the origin of the genes that encode viral antigens is therefore of vital importance.

The influenza virus is traditionally characterized at the molecular level using the reverse transcriptase polymerase chain reaction (RT-PCR). The isolation of viral RNA is followed by reverse transcription of prescribed gene segments to generate cDNA, their subsequent amplification using primers to these targets, and the detection and/or sequencing of the amplificons. Strategies to characterize reassortant strains of the virus have been developed [9], [10], but since gene reassortment appears to be random, full characterization of a progeny virus requires the sequencing of part or all of the eight genes, a process that is quite laborious and can take several days to achieve.

A rapid and direct proteotyping approach with which to both type and subtype influenza viral strains employing proteomics methods and high resolution mass spectrometry has recently been reported for seasonal strains [11]–[14]. The detection of a single signature peptide ion representing a conserved sequence of a viral antigen that is also unique in mass, within a few part-per-million (ppm), when compared to the *in silico* digestion products of all influenza antigens from all hosts, is sufficient to be able to confidently type and subtype strains of the influenza virus. It complements related studies that have employed proteomics methods and mass spectrometry to characterise the antigenicity of the influenza virus [15]–[18]. More recently, it has been shown that the proteotyping approach can distinguish seasonal from pandemic type A H1N1 influenza strains [19] and also assign the lineage of human strains of type A H1N1 and type B influenza virus [20].

Given the pandemic potential of reassorted strains of influenza virus, this study demonstrates that the origin of the genes encoding the viral antigens within reassorted strains can also be rapidly and unequivocally determined using this proteotyping strategy. Establishing the gene origin of reassorted pandemic strains of the virus is essential to the design and production of vaccines against such strains and to monitor their evolutionary history. This is illustrated a strain formed by the reassortment of a wild-type and an egg adapted high-growth strain that was constructed for a vaccine that has been administrated against 2009 pandemic H1N1 influenza virus.

## Results and Discussion

**Figure 1:**
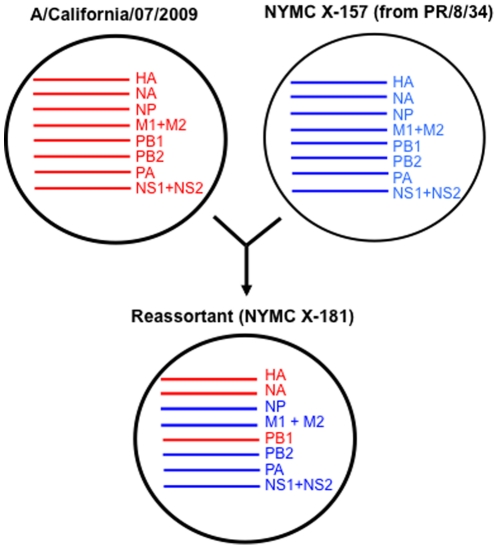
Representation of the gene origin of the reassortant (NYMC X-181) strain used to vaccinate against 2009 pandemic H1N1 influenza.

The PanVax H1N1 vaccine, produced against pandemic 2009 H1N1 influenza (“swine flu”), contains a single strain of influenza virus without adjuvant. The vaccine was prepared from a classical genetic reassortment of a wild-type strain (A/California/07/2009) and an egg adapted high-growth strain (NYMC X-157) derived from an earlier A/Puerto Rico/8/34 strain. The reassorted vaccine strain thus contains genes that originated from both source strains (Figure 1).

**Figure 2:**
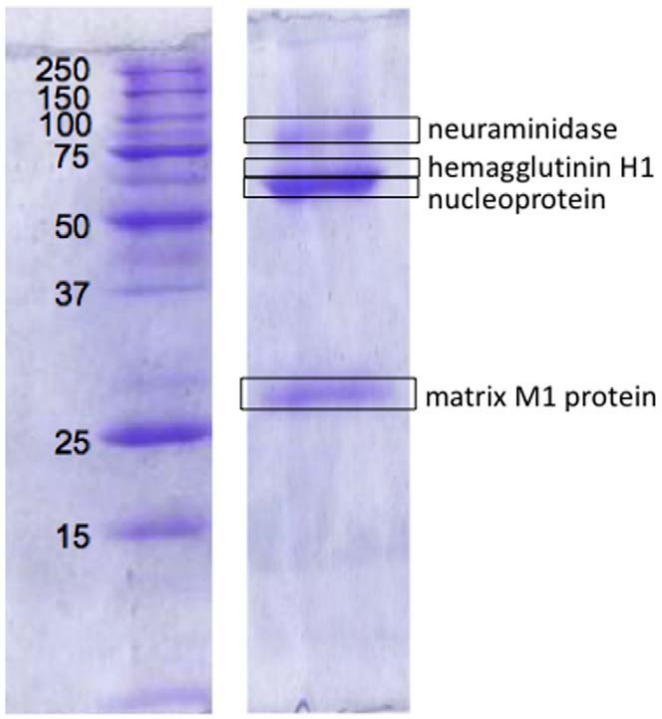
SDS-PAGE gel showing the viral antigens neuraminidase, hemagglutinin H1 subunit, nucleoprotein and matrix M1 protein of the reassortant (NYMC X-181) strain aside molecular weight markers.

The major antigens derived from the vaccine strain were partially separated by SDS-PAGE (Figure 2). The most prominent bands were detected for nucleoprotein and matrix M1 protein in accord with their higher copy numbers per virion, as evident in our previous studies [18], [19]. The matrix protein constitutes some 40% of the total viral protein while the hemagglutinin and neuraminidase surface antigens constitute only 25% and 5% of all viral protein respectively.

**Figure 3:**
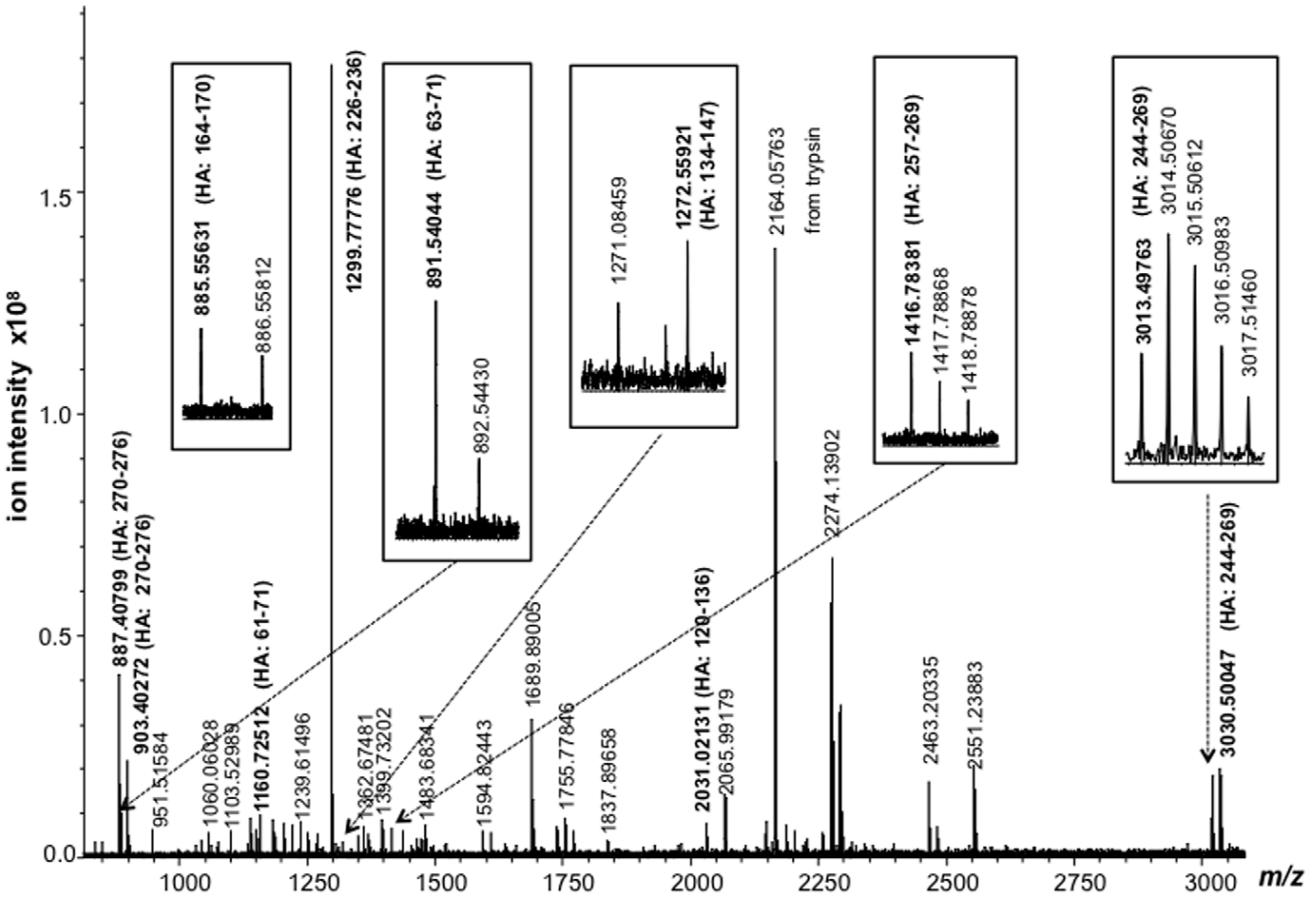
High resolution MALDI mass spectrum of the tryptic digest of the hemagglutinin H1 antigen (HA) band of **Figure 2**.

The hemagglutinin antigen appears as a partially resolved, faint band above that of nucleoprotein at a molecular weight of approximately 65 kDa. The high resolution MALDI mass spectrum of this band is shown in Figure 3. Eleven of the tryptic peptides detected (labelled in bold) are associated with the hemagglutinin antigen. With the exception of one peptide, all are unique in sequence to those derived from the A/California/7/2009 strain correctly establishing it as the origin of the hemagglutinin gene within the reassorted vaccine strain. A single peptide at *m/z* 2030.0213 comprises HA residues 120–136 which has an identical sequence in both the A/California/7/2009 and A/Puerto Rico/8/34 strain.

The MALDI spectrum also enables the strain to be identified as the vaccine strain, over the originating A/California/7/2009 strain, due to the detection of a dominant peptide ion of unique sequence that comprises HA residues 226–236 (TFKPEIAIRPK) at *m/z* 1299.7778. The threonine residue at position 226 replaces lysine in the original A/California/7/2009 strain and thus increases the peptide's mass by 27 units.

**Figure 4:**
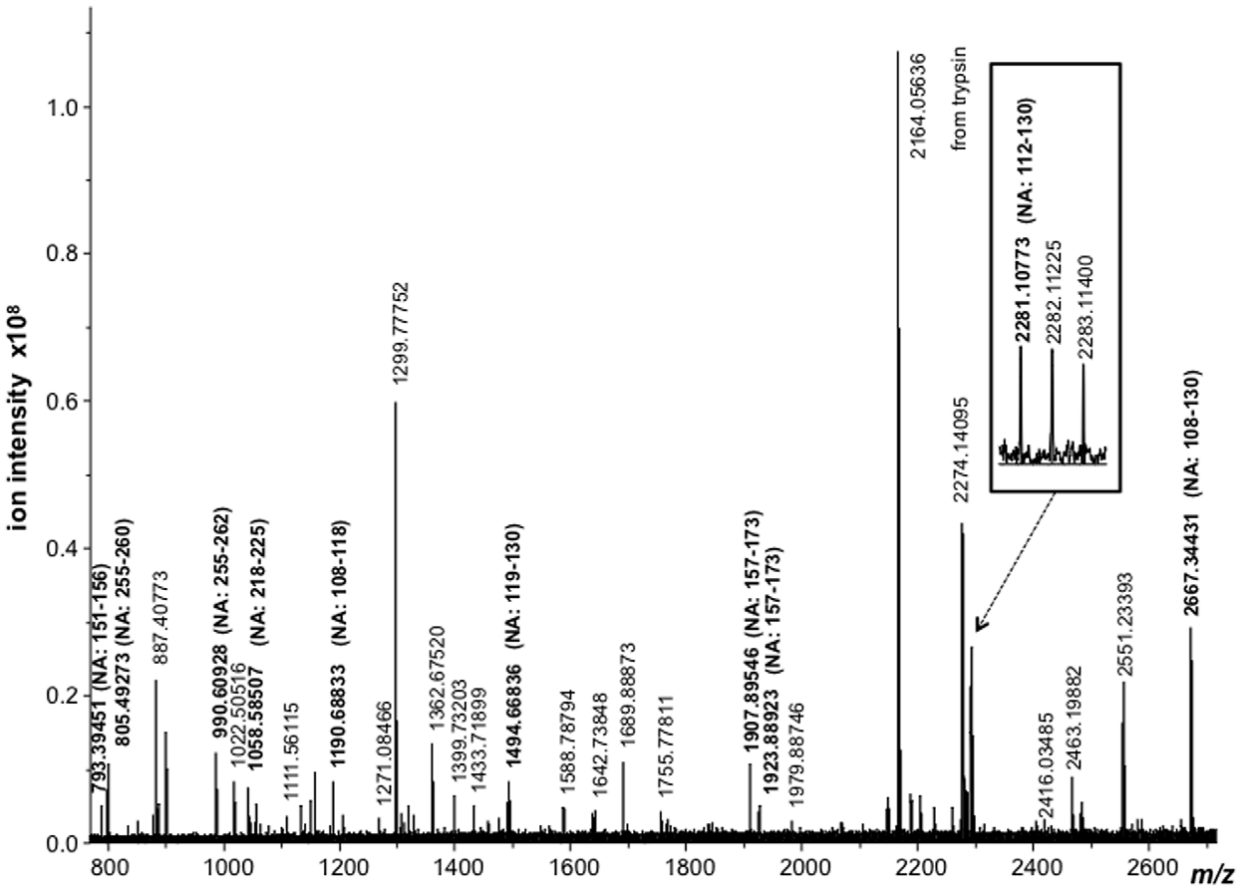
High resolution MALDI mass spectrum of the tryptic digest of the neuraminidase antigen (NA) band of **Figure 2**.

The high resolution MALDI mass spectrum of the upper neuraminidase band of Figure 2 is shown in Figure 4. This spectrum exhibits 10 tryptic peptides derived from the neuraminidase antigen of the A/California/7/2009 strain that enable this strain to be identified as the origin of the NA gene. In this case, two peptides comprising residues HA 151–156 and 108–118 at *m/z* 793.3945 and 1190.6883 share a common sequence and mass with the A/Puerto Rico/8/34 strain. The remaining 8 are unique to the A/California/7/2009 strain.

Both surface antigens, hemagglutinin and neuraminidase, are identified to be derived from the A/California/7/2009 strain. The reverse is true in the case of the nucleoprotein (NP) and matrix protein 1 (M1). The tryptic peptides that were detected following the digestion of the NP and M1 bands, at approximately 65 and 30kDa (in Figure 2), are shown in Tables S1 and S2 respectively. The larger number of peptide ions detected for these antigens is associated with the higher copy numbers of these antigens per virion reflected in the more predominant bands on the gel.

In the case of the nucleoprotein antigen, the detected peptides represent 59% of the total sequence. While some NP peptides share a common sequence in the nucleoprotein sequence of the A/California/7/2009 and A/Puerto Rico/8/34 strains, the majority (shaded) are unique in sequence to the A/Puerto Rico/8/34 strain (38% coverage) and enable the origin of the gene in the reassortant strain to be correctly assigned to this strain.

Of the 39 NP peptides detected, three represent type A signature peptides for this antigen [12] at *m/z* 672.4422, 961.4442 and 1067.5194 enable the reassortant virus strain to be unambiguously assigned as a type A strain. Furthermore, two H1N1 subtype specific signature peptides are detected at *m/z* 1344.5345 and 2036.9376 [12]. The latter represents the modified form of the peptide comprised of NP residues 326–342 in which the methinione residue is oxidised and the cysteine residue is carbamidomethylated due to the reduction and alkylation of cysteine residues prior to tryptic digestion.

In the case of the matrix protein 1 (M1), 29 tryptic peptides are detected spanning 77% of the total protein sequence. Those shaded have a sequence, and thus mass, that is unique to the A/Puerto Rico/8/34 strain. Their detection enables the origin of the matrix protein gene to be established as the A/Puerto Rico/8/34 strain. Eight of the tryptic peptides (labelled at their sequence in Table S2) represent or contain type A matrix M1 protein signature peptides that further confirm the virus strain type [13].

**Figure 5:**
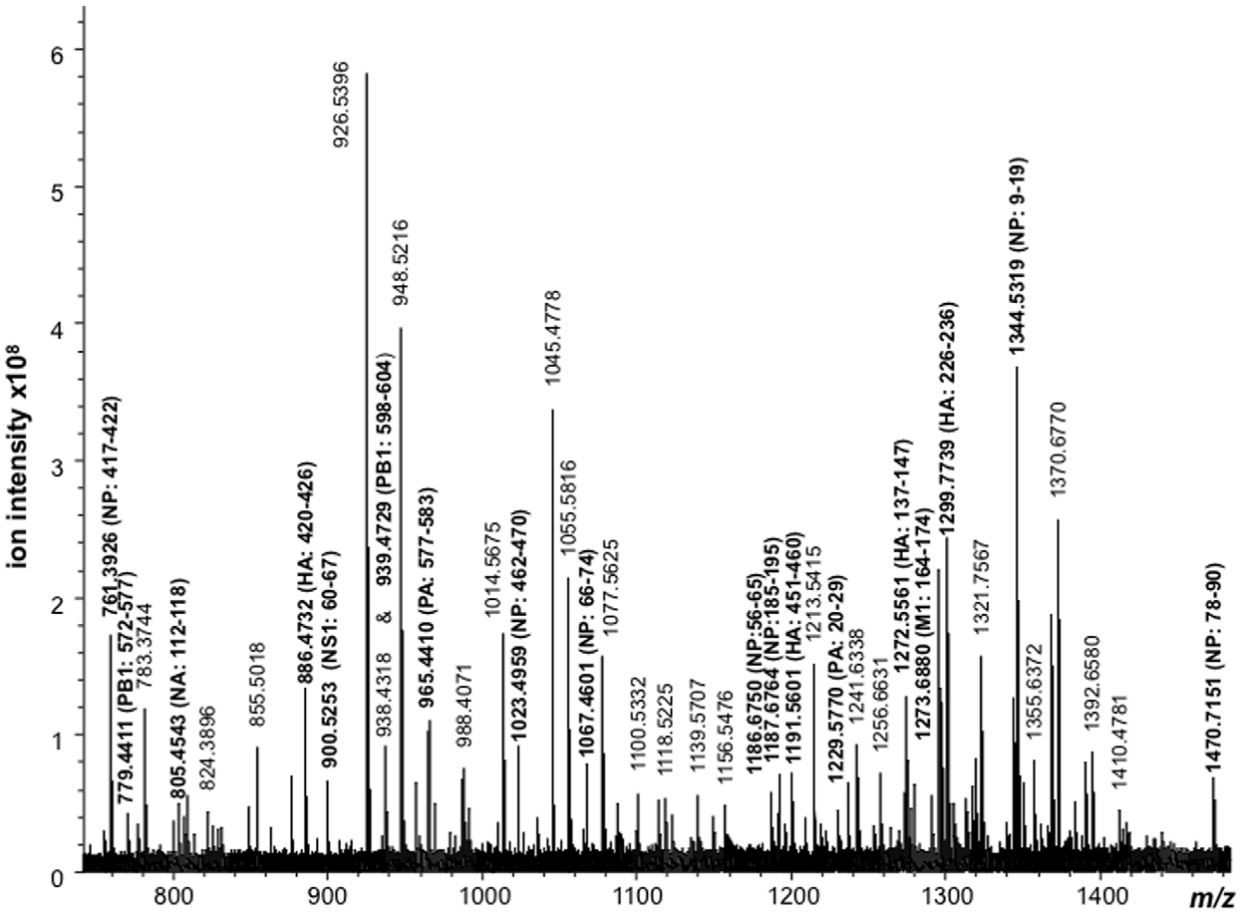
High resolution MALDI mass spectrum of the tryptic digest of the whole PanVax H1N1 vaccine.

To establish that the proteotyping approach can also be applied to identify gene origin in whole virus, without the need to separate the component viral antigens, a solution of digested whole vaccine was analyzed. The MALDI mass spectrum of the tryptic products generated is shown in Figure 5. Although fewer peptide ions are detected, due both to the steric constraints imposed by the virion that impedes digestion and the competitive ionisation processes when large mixtures of peptides are analysed, all four antigens are represented in the spectrum. Among the hemagglutinin peptides detected, the peptide comprised of residues 226–236 (at *m/z* 1299.7739) described above establishes the origin of the HA gene to be the A/California/7/2009 strain. A sole NA peptide at *m/z* 805.4543 comprises residues 112–118 of the antigen also has a sequence (GDVFVIR) unique to the A/California/7/2009 strain.

Of the nucleoprotein peptide ions detected, those at *m/z* 761.3926, 1067.4601, 1187.6764 and 1344.5319 are all unique to the A/Puerto Rico/8/34 strain. The M1 peptide detected at *m/z* 1273.6880 corresponding to residues 164–174 is also of a sequence that is unique to the reassortant strain. This confirms the A/Puerto Rico/8/34 strain is the origin of both the matrix and nucleoprotein, though the reassortant strain and not the originating strain is that which has been analysed.

Although not detected by SDS-PAGE (Figure 2) due to their lower levels (100–3000 fold less) per virion, the viral polymerases (PA and PB) and non-structural proteins (NS) were detected within the spectrum of the whole vaccine digest (Figure 5). For examples, ions detected at *m/z* 965.4410, 1229.5770 and 1278.6437 are associated with residues 577–583, 20–29 and 246–256 of polymerase PA while ions at *m/z* 779.4411, 877.4852 and 939.4729 are associated with residues 572–577 and 472–479 of polymerase PB1, and residues 598–604 of polymerase PB2, respectively. However, since these proteins are highly conserved among strains and share a 96% sequence identity and 99% sequence homology across the A/California/7/2009 and A/Puerto Rico/8/34 strains they provide little to no value for establishing the origin of their genes.

The application of a proteotyping approach, previously applied to the rapid and direct identification of the type, subtype and lineage of influenza strains, has been successfully utilized here to identify the origin of genes within a reassorted strain of the influenza virus. Given the these reassorted strains pose the greatest risk to human and animal health and have been responsible for all past human pandemics of the 20th and 21st centuries, the ability to rapidly and reliably establish the origins and evolutionary history of such strains at a molecular level is of paramount importance. In the case of whole virus digests, the approach is limited only by the time required for proteolytic digestion, a process that can be accelerated using microwave irradiation [21]. Due to the nature of the MALDI target plate, hundreds (typically 100–384) of virus samples, digested in parallel, can be applied to a single plate with a mass spectrum for each sample acquired within fractions of a seconds to a few minutes. The approach does require access to a high resolution mass spectrometer of some considerable cost (typically $US500K-1M) but has broader applicability to other biopathogens that threaten human and animal health.

## Materials and Methods

### Influenza virus reassortment strain

The PanVax H1N1 vaccine was donated by CSL Biotherapies, CSL Limited (Parkville, Victoria, Australia) and used without further purification. The vaccine is formulated at a dose corresponding to 30 ng/mL of hemagglutinin. It contains strain NYMC X-181, a reassortant strain comprised of genomic segments coding for the HA, NA and PB1 antigens which were derived from the pandemic (H1N1) 2009 strain A/California/7/2009. The remaining segments are derived from the A/Puerto Rico/8/34 (H1N1) strain. Translated protein sequences were derived from GenBank [22] via the Influenza Virus Resource [23] with the following accession numbers: HA ACV82259 (reassortant), ACP44189 (A/California/7/2009); NA ACV82260 (reassortant), ACT36688 (A/California/7/2009); NP ADE29086 (reassortant), CAA24268 (A/Puerto Rico/8/34); M1 ADE29088 (reassortant), ABD77676 (A/Puerto Rico/8/34).

### Viral antigen recovery

The PanVax H1N1 vaccine (120 µL containing 10 ng of total viral antigen) was subjected to SDS-PAGE using a Bio-Rad TetraCell gel box attached to a Bio-Rad Powerpac 300 power supply (Bio-Rad, Hercules, CA, USA). The vaccine was concentrated to approximately 10 µL by a vacuum concentrator (Labconco Corporation, Kansas City, MI, USA) and diluted in 1∶2 with Laemmli buffer containing 100mM Tris-HCl (pH 6.8), 10% (v/v) glycerol, 2% (w/v) SDS, 3.3% (v/v) 2-mercaptoethanol and 0.02mg/ml Bromophenol Blue. The sample was then heated for 10 min at 97°C in an Eppendorf Thermomixer 5468 (Eppendorf, Hamburg, Germany). A Precision Plus protein standard solution (Bio-Rad, Hercules, CA, USA) (15 µL) was loaded alongside the vaccine sample on a polyacrylamide gel (12.5% separating and 5% stacking gels). SDS-PAGE was performed in a buffered solution containing 25 mM Tris-HCl, 192 mM glycine and 0.1% (w/v) SDS (pH 7.5) at 15 mA for 1.5 hours. The separated bands were visualised by a Coomassie Brilliant Blue solution (0.25% w/v Coomassie Brilliant Blue, 45% v/v methanol and 10% v/v glacial acetic acid). The gel was then destained in an aqueous solution containing 40% v/v methanol and 7% v/v glacial acetic acid for 1 hour and subsequently in an aqueous solution containing 5% v/v methanol and 7% v/v glacial acetic acid overnight. The gel was scanned using a Bio-Rad, GS-800 densitometer (Bio-Rad, Hercules, CA, USA).

### In-gel tryptic digestion

The bands containing hemagglutinin, neuraminidase, nucleoprotein and matrix M1 protein were manually excised and reconstituted in a solution of 25 mM ammonium bicarbonate (NH_4_HCO_3_) in 50% v/v acetonitrile (ACN) at 37°C. The cysteine residues were reduced with dithiothreitol (DTT) (10 mM in 50 mM NH_4_HCO_3_ pH 8.5) for 30 min at 56°C and alkylated with iodoacetamide (55 mM iodoacetamide in 50 mM NH_4_HCO_3_ pH 8.5) for 20 min at room temperature with protection from ambient light. Excess iodoacetamide was removed by washing with ACN (3×350 µL) and the gel pieces were dried in a vacuum concentrator (Labconco Corporation, Kansas City, MI, USA). In-gel tryptic digestion of the hemagglutinin, neuraminidase, nucleoprotein and matrix M1 protein was performed using 13 ng·µL^−1^ of modified trypsin (Roche Diagnostics GmbH, Mannheim, Germany) overnight at 37°C in a digestion buffer containing 25 mM NH_4_HCO_3_, 10% ACN and 3.4 mM octyl-β-D-glucopyranoside. The resulting peptides were extracted by repeated sonication in 60% ACN containing 0.1% trifluoroacetic acid (TFA). The extracted peptides were dried completely in a vacuum concentrator and redissolved in a 25 mM NH_4_HCO_3_ solution.

### Whole vaccine tryptic digestion

A suspension corresponding to 35µg of the whole virus was concentrated to near dryness in a vacuum concentrator and redissolved in 50µL digestion buffer (50 mM NH_4_HCO_3_, 10% ACN and 2 mM DTT, pH 7.5). Sequence-grade modified trypsin (Roche Diagnostics GmbH, Mannheim, Germany) was added at 13ng·µL^−1^ and the solution was incubated overnight at 37°C.

### High resolution MALDI mass spectrometry

Each digested solution (1 µL) was diluted with in a solution (3 µL) of a MALDI matrix (2 mg·mL^−1^ α-cyano-4-hydroxycinnaminic acid in 50% ACN containing 0.1% TFA). The mixed analyte and matrix solution (1 µL) was spotted onto a MALDI sample plate (MTP AnchorChip™ 400/384 TF, Bruker Daltonics, Billerica, MA, USA) and dried by evaporation in air. MALDI FT-ICR mass spectra were recorded on a 7T Bruker APEX-Qe instrument (Bruker Daltonics, Billerica, MA, USA) in the positive ion mode. Ions released with between 25–200 laser shots (at 5% laser power) from the MALDI plate, held at 400V, were accumulated above the plate for 0.2 sec, stored in the hexapole for 1.0 sec, and then passed to the FT-ICR cell in 1.0 msec using a side kick voltage of 0 V and an offset of −1.5 V. 15–32 scans were acquired and averaged into a single mass spectrum. Spectra were acquired with 1M data points using a broadband excitation across a mass range of *m/z* 404–4000. A mass resolution of over 100,000 (FWHM) at *m/z* 1296 was typically achieved. The instrument was mass calibrated with an external mixture of peptides comprising Angiotensin I, adrenocorticotropic hormone (ACTH) fragments comprising residues 1–17, 7–38 and 18–39, and a synthetic hemagglutinin antigen derived peptide. Mass spectra were processed using the Data Analysis v3.4 software (Billerica, MA, USA) and, in some cases, internally mass calibrated using identified peptide ions in each spectrum derived from the viral proteins or tryptic autolysis products. Peptides were identified through a search of the NCBI non-redundant protein database using the Mascot Peptide Mass Fingerprint algorithm [24] with the following constraints: taxonomy – viruses, modifications - oxidation (M), Gln/Glu to Pyro-Glu, and carbomidomethyl-cysteine, and a peptide tolerance of +/−5 ppm. These searches were performed in conjunction with manual assignments of peptide ions using theoretical monoisotopic mass values for tryptic digest products of database antigen sequences generated by the MS-Fit algorithm [25] using the same criteria. Mass accuracies of better than 5ppm were routinely achieved for all ions detected.

## Supporting Information

Table S1 Nucleoprotein derived peptides from the tryptic digestion of the NP band of the NYMC X-181 reassortant strain as detected by MALDI-MS.
(DOC)

Table S2 Matrix M1 protein derived peptides from the tryptic digestion of the M1 band of the NYMC X-181 reassortant strain as detected by MALDI-MS.
(DOC)
