# Supplementary material for: Proteotyping to Establish Gene Origin within Reassortant Influenza Viruses
Source: PLoS One. 2011 Jan 31;6(1):e15771. doi: 10.1371/journal.pone.0015771 (PMC3031537; doi:10.1371/journal.pone.0015771)
Supplement: Table S1 — Nucleoprotein derived peptides from the tryptic digestion of the NP band of the NYMC X-181 reassortant strain as detected by MALDI-MS. (DOC) [file pone.0015771.s001.doc]

Table S1 Nucleoprotein derived peptides from the tryptic digestion of the NP band of the NYMC X-181 reassortant strain as detected by MALDI-MS

| **m/z [M+H]+**  **monoisotopic**  **(experimental)** | **Δ (ppm)** | **residues** | **missed cleavage sites** | **amino acid sequence** | **modifications*** |
| --- | --- | --- | --- | --- | --- |
| 672.4422 | 2.84 | 262-267 | 0 | [SALILR](http://prospector.ucsf.edu/prospector/cgi-bin/mssearch.cgi?search_name=msproduct&output_type=HTML&report_title=MS-Product&version=5.5.0&instrument_name=MALDI-Q-TOF&use_instrument_ion_types=1&parent_mass_convert=monoisotopic&user_aa_composition=C2 H3 N1 O1&max_charge=1&sequence=SALILR&) ** |  |
| 708.3846 | 2.54 | 385-389 | 0 | [YWAIR](http://prospector.ucsf.edu/prospector/cgi-bin/mssearch.cgi?search_name=msproduct&output_type=HTML&report_title=MS-Product&version=5.5.0&instrument_name=MALDI-Q-TOF&use_instrument_ion_types=1&parent_mass_convert=monoisotopic&user_aa_composition=C2 H3 N1 O1&max_charge=1&sequence=YWAIR&) |  |
| **761.3950** | **1.18** | **417-422** | **0** | [**NLPFDR**](http://prospector.ucsf.edu/prospector/cgi-bin/mssearch.cgi?search_name=msproduct&output_type=HTML&report_title=MS-Product&version=5.5.0&instrument_name=MALDI-Q-TOF&use_instrument_ion_types=1&parent_mass_convert=monoisotopic&user_aa_composition=C2 H3 N1 O1&max_charge=1&sequence=NLPFDR&) |  |
| 850.3913 | 0.29 | 237-243 | 0 | [AMMDQVR](http://prospector.ucsf.edu/prospector/cgi-bin/mssearch.cgi?search_name=msproduct&output_type=HTML&report_title=MS-Product&version=5.5.0&instrument_name=MALDI-Q-TOF&use_instrument_ion_types=1&parent_mass_convert=monoisotopic&user_aa_composition=C2 H3 N1 O1&max_charge=1&sequence=AMMDQVR&) |  |
| 891.5061 | 1.57 | 91-98 | 1 | [KTGGPIYR](http://prospector.ucsf.edu/prospector/cgi-bin/mssearch.cgi?search_name=msproduct&output_type=HTML&report_title=MS-Product&version=5.5.0&instrument_name=MALDI-Q-TOF&use_instrument_ion_types=1&parent_mass_convert=monoisotopic&user_aa_composition=C2 H3 N1 O1&max_charge=1&sequence=KTGGPIYR&) |  |
| **908.4947** | **-0.17** | **215-221** | **1** | [**TRIAYER**](http://prospector.ucsf.edu/prospector/cgi-bin/mssearch.cgi?search_name=msproduct&output_type=HTML&report_title=MS-Product&version=5.5.0&instrument_name=MALDI-Q-TOF&use_instrument_ion_types=1&parent_mass_convert=monoisotopic&user_aa_composition=C2 H3 N1 O1&max_charge=1&sequence=TRIAYER&) **#** |  |
| 919.5112 | 0.44 | 92-99 | 1 | [TGGPIYRR](http://prospector.ucsf.edu/prospector/cgi-bin/mssearch.cgi?search_name=msproduct&output_type=HTML&report_title=MS-Product&version=5.5.0&instrument_name=MALDI-Q-TOF&use_instrument_ion_types=1&parent_mass_convert=monoisotopic&user_aa_composition=C2 H3 N1 O1&max_charge=1&sequence=TGGPIYRR&) |  |
| 951.5162 | 0.34 | 383-389 | 1 | [SRYWAIR](http://prospector.ucsf.edu/prospector/cgi-bin/mssearch.cgi?search_name=msproduct&output_type=HTML&report_title=MS-Product&version=5.5.0&instrument_name=MALDI-Q-TOF&use_instrument_ion_types=1&parent_mass_convert=monoisotopic&user_aa_composition=C2 H3 N1 O1&max_charge=1&sequence=SRYWAIR&) # |  |
| **961.4442** | **-0.37** | **392-400** | **0** | [**SGGNTNQQR**](http://prospector.ucsf.edu/prospector/cgi-bin/mssearch.cgi?search_name=msproduct&output_type=HTML&report_title=MS-Product&version=5.5.0&instrument_name=MALDI-Q-TOF&use_instrument_ion_types=1&parent_mass_convert=monoisotopic&user_aa_composition=C2 H3 N1 O1&max_charge=1&sequence=SGGNTNQQR&) ****** |  |
| **1067.5194** | **0.33** | **66-74** | **0** | [**MVLSAFDER**](http://prospector.ucsf.edu/prospector/cgi-bin/mssearch.cgi?search_name=msproduct&output_type=HTML&report_title=MS-Product&version=5.5.0&instrument_name=MALDI-Q-TOF&use_instrument_ion_types=1&parent_mass_convert=monoisotopic&user_aa_composition=C2 H3 N1 O1&max_charge=1&sequence=MVLSAFDER&) ****** | **also as Mox** |
| 1135.5393 | 0.04 | 205-213 | 1 | [NFWRGENGR](http://prospector.ucsf.edu/prospector/cgi-bin/mssearch.cgi?search_name=msproduct&output_type=HTML&report_title=MS-Product&version=5.5.0&instrument_name=MALDI-Q-TOF&use_instrument_ion_types=1&parent_mass_convert=monoisotopic&user_aa_composition=C2 H3 N1 O1&max_charge=1&sequence=NFWRGENGR&) |  |
| 1177.5865 | 0.32 | 200-208 | 1 | [GINDRNFWR](http://prospector.ucsf.edu/prospector/cgi-bin/mssearch.cgi?search_name=msproduct&output_type=HTML&report_title=MS-Product&version=5.5.0&instrument_name=MALDI-Q-TOF&use_instrument_ion_types=1&parent_mass_convert=monoisotopic&user_aa_composition=C2 H3 N1 O1&max_charge=1&sequence=GINDRNFWR&) |  |
| 1186.6791 | 0.05 | 56-65 | 0 | [LIQNSLTIER](http://prospector.ucsf.edu/prospector/cgi-bin/mssearch.cgi?search_name=msproduct&output_type=HTML&report_title=MS-Product&version=5.5.0&instrument_name=MALDI-Q-TOF&use_instrument_ion_types=1&parent_mass_convert=monoisotopic&user_aa_composition=C2 H3 N1 O1&max_charge=1&sequence=LIQNSLTIER&) |  |
| **1191.6229** | **0.40** | **185-195** | **0** | [**GVGTMVMELVR**](http://prospector.ucsf.edu/prospector/cgi-bin/mssearch.cgi?search_name=msproduct&output_type=HTML&report_title=MS-Product&version=5.5.0&instrument_name=MALDI-Q-TOF&use_instrument_ion_types=1&parent_mass_convert=monoisotopic&user_aa_composition=C2 H3 N1 O1&max_charge=1&sequence=GVGTMVMELVR&) | **also as Mox** |
| **1218.5929** | **-0.42** | **390-400** | **1** | [**TRSGGNTNQQR**](http://prospector.ucsf.edu/prospector/cgi-bin/mssearch.cgi?search_name=msproduct&output_type=HTML&report_title=MS-Product&version=5.5.0&instrument_name=MALDI-Q-TOF&use_instrument_ion_types=1&parent_mass_convert=monoisotopic&user_aa_composition=C2 H3 N1 O1&max_charge=1&sequence=TRSGGNTNQQR&) |  |
| **1221.6257** | **0.03** | **437-446** | **1** | [**TSDMRTEIIR**](http://prospector.ucsf.edu/prospector/cgi-bin/mssearch.cgi?search_name=msproduct&output_type=HTML&report_title=MS-Product&version=5.5.0&instrument_name=MALDI-Q-TOF&use_instrument_ion_types=1&parent_mass_convert=monoisotopic&user_aa_composition=C2 H3 N1 O1&max_charge=1&sequence=TSDMRTEIIR&) | **also as Mox** |
| 1223.6209 | 0.61 | 66-75 | 1 | [MVLSAFDERR](http://prospector.ucsf.edu/prospector/cgi-bin/mssearch.cgi?search_name=msproduct&output_type=HTML&report_title=MS-Product&version=5.5.0&instrument_name=MALDI-Q-TOF&use_instrument_ion_types=1&parent_mass_convert=monoisotopic&user_aa_composition=C2 H3 N1 O1&max_charge=1&sequence=MVLSAFDERR&) | also in Mox |
| **1344.5345** | **-2.04** | **9-19** | **0** | [**SYEQMETDGER**](http://prospector.ucsf.edu/prospector/cgi-bin/mssearch.cgi?search_name=msproduct&output_type=HTML&report_title=MS-Product&version=5.5.0&instrument_name=MALDI-Q-TOF&use_instrument_ion_types=1&parent_mass_convert=monoisotopic&user_aa_composition=C2 H3 N1 O1&max_charge=1&sequence=SYEQMETDGER&) **#** |  |
| **1352.6840** | **-0.36** | **294-305** | **0** | [**EGYSLVGIDPFR**](http://prospector.ucsf.edu/prospector/cgi-bin/mssearch.cgi?search_name=msproduct&output_type=HTML&report_title=MS-Product&version=5.5.0&instrument_name=MALDI-Q-TOF&use_instrument_ion_types=1&parent_mass_convert=monoisotopic&user_aa_composition=C2 H3 N1 O1&max_charge=1&sequence=EGYSLVGIDPFR&) | **also as PyroGlu** |
| 1380.6440 | 0.58 | 163-174 | 0 | MCSLMQGSTLPR | only as  Mox, 2xMox, Carbamido |
| 1470.7229 | 0.42 | 78-90 | 1 | [YLEEHPSAGKDPK](http://prospector.ucsf.edu/prospector/cgi-bin/mssearch.cgi?search_name=msproduct&output_type=HTML&report_title=MS-Product&version=5.5.0&instrument_name=MALDI-Q-TOF&use_instrument_ion_types=1&parent_mass_convert=monoisotopic&user_aa_composition=C2 H3 N1 O1&max_charge=1&sequence=YLEEHPSAGKDPK&) |  |
| **1482.6999** | **-0.50** | **423-436** | **0** | [**TTIMAAFNGNTEGR**](http://prospector.ucsf.edu/prospector/cgi-bin/mssearch.cgi?search_name=msproduct&output_type=HTML&report_title=MS-Product&version=5.5.0&instrument_name=MALDI-Q-TOF&use_instrument_ion_types=1&parent_mass_convert=monoisotopic&user_aa_composition=C2 H3 N1 O1&max_charge=1&sequence=TTIMAAFNGNTEGR&) |  |
| 1536.7453 | 0.63 | 163-175 | 1 | MCSLMQGSTLPRR | only as  Mox, 2xMox, Carbamido |
| 1598.8193 | 1.25 | 78-91 | 2 | [YLEEHPSAGKDPKK](http://prospector.ucsf.edu/prospector/cgi-bin/mssearch.cgi?search_name=msproduct&output_type=HTML&report_title=MS-Product&version=5.5.0&instrument_name=MALDI-Q-TOF&use_instrument_ion_types=1&parent_mass_convert=monoisotopic&user_aa_composition=C2 H3 N1 O1&max_charge=1&sequence=YLEEHPSAGKDPKK&) |  |
| **1689.8918** | **-0.04** | **401-416** | **0** | [**ASAGQISIQPTFSVQR**](http://prospector.ucsf.edu/prospector/cgi-bin/mssearch.cgi?search_name=msproduct&output_type=HTML&report_title=MS-Product&version=5.5.0&instrument_name=MALDI-Q-TOF&use_instrument_ion_types=1&parent_mass_convert=monoisotopic&user_aa_composition=C2 H3 N1 O1&max_charge=1&sequence=ASAGQISIQPTFSVQR&) |  |
| **1693.8186** | **0.38** | **247-261** | **0** | [**NPGNAEFEDLTFLAR**](http://prospector.ucsf.edu/prospector/cgi-bin/mssearch.cgi?search_name=msproduct&output_type=HTML&report_title=MS-Product&version=5.5.0&instrument_name=MALDI-Q-TOF&use_instrument_ion_types=1&parent_mass_convert=monoisotopic&user_aa_composition=C2 H3 N1 O1&max_charge=1&sequence=NPGNAEFEDLTFLAR&) |  |
| 1712.8568 | -1.99 | 76-90 | 2 | [NKYLEEHPSAGKDPK](http://prospector.ucsf.edu/prospector/cgi-bin/mssearch.cgi?search_name=msproduct&output_type=HTML&report_title=MS-Product&version=5.5.0&instrument_name=MALDI-Q-TOF&use_instrument_ion_types=1&parent_mass_convert=monoisotopic&user_aa_composition=C2 H3 N1 O1&max_charge=1&sequence=NKYLEEHPSAGKDPK&) |  |
| **1739.7866** | **1.49** | **447-461** | **0** | [**MMESARPEDVSFQGR**](http://prospector.ucsf.edu/prospector/cgi-bin/mssearch.cgi?search_name=msproduct&output_type=HTML&report_title=MS-Product&version=5.5.0&instrument_name=MALDI-Q-TOF&use_instrument_ion_types=1&parent_mass_convert=monoisotopic&user_aa_composition=C2 H3 N1 O1&max_charge=1&sequence=MMESARPEDVSFQGR&) | **also as  Mox, 2xMox** |
| 2036.9376 | 2.91 | 326-342 | 0 | SQLVWMACHSAAFEDLR # | only as Mox,  Carbamido |
| 2053.9336 | 2.45 | 157-174 | 1 | TGMDPRMCSLMQGSTLPR | only as  Mox, 2xMox, Carbamido |
| **2065.9983** | **2.17** | **244-261** | **1** | [**ESRNPGNAEFEDLTFLAR**](http://prospector.ucsf.edu/prospector/cgi-bin/mssearch.cgi?search_name=msproduct&output_type=HTML&report_title=MS-Product&version=5.5.0&instrument_name=MALDI-Q-TOF&use_instrument_ion_types=1&parent_mass_convert=monoisotopic&user_aa_composition=C2 H3 N1 O1&max_charge=1&sequence=ESRNPGNAEFEDLTFLAR&) | **also as PyroGlu** |
| **2156.9559** | **2.13** | **9-26** | **1** | [**SYEQMETDGERQNATEIR**](http://prospector.ucsf.edu/prospector/cgi-bin/mssearch.cgi?search_name=msproduct&output_type=HTML&report_title=MS-Product&version=5.5.0&instrument_name=MALDI-Q-TOF&use_instrument_ion_types=1&parent_mass_convert=monoisotopic&user_aa_composition=C2 H3 N1 O1&max_charge=1&sequence=SYEQMETDGERQNATEIR&) | **also as Mox** |
| 2169.0065 | 1.53 | 157-175 | 2 | TGMDPRMCSLMQGSTLPRR | only as 2xMox |
| **2321.2416** | **2.35** | **306-325** | **0** | [**LLQNSQVYSLIRPNENPAHK**](http://prospector.ucsf.edu/prospector/cgi-bin/mssearch.cgi?search_name=msproduct&output_type=HTML&report_title=MS-Product&version=5.5.0&instrument_name=MALDI-Q-TOF&use_instrument_ion_types=1&parent_mass_convert=monoisotopic&user_aa_composition=C2 H3 N1 O1&max_charge=1&sequence=LLQNSQVYSLIRPNENPAHK&) |  |
| **2329.0543** | **3.01** | **8-26** | **2** | **RSYEQMETDGERQNATEIR** | **only as Mox** |
| **2339.0910** | **2.40** | **362-382** | **0** | [**GVQIASNENMETMESSTLELR**](http://prospector.ucsf.edu/prospector/cgi-bin/mssearch.cgi?search_name=msproduct&output_type=HTML&report_title=MS-Product&version=5.5.0&instrument_name=MALDI-Q-TOF&use_instrument_ion_types=1&parent_mass_convert=monoisotopic&user_aa_composition=C2 H3 N1 O1&max_charge=1&sequence=GVQIASNENMETMESSTLELR&) | **also as Mox** |
| **2368.1474** | **3.78** | **442-461** | **1** | **TEIIRMMESARPEDVSFQGR** | **only as Mox** |
| **2582.2209** | **0.94** | **362-384** | **1** | [**GVQIASNENMETMESSTLELRSR**](http://prospector.ucsf.edu/prospector/cgi-bin/mssearch.cgi?search_name=msproduct&output_type=HTML&report_title=MS-Product&version=5.5.0&instrument_name=MALDI-Q-TOF&use_instrument_ion_types=1&parent_mass_convert=monoisotopic&user_aa_composition=C2 H3 N1 O1&max_charge=1&sequence=GVQIASNENMETMESSTLELRSR&) |  |
| **2990.3794** | **0.95** | **437-461** | **2** | **TSDMRTEIIRMMESARPEDVSFQGR** | **only as 3xMox** |

- Modifications Mox, 2xMox, PyroGlu and carbamido denote the oxidationand dioxidation of methionine residues, the presence of a pyroglutamic acid residue, and the carbamidomethyl alkylation of a cysteine residue respectively.

** Denotes a nucleoprotein type A signature peptide, or a peptide containing a nucleoprotein signature, within 2009 H1N1 pandemic influenza virus (Schwahn et al., 2009b)

# Denotes a nucleoprotein type A signature peptide specific to human H1N1 subtype

Peptides in bold are only found in A/PuertoRico/8/34 originating strain
