# Supplementary material for: Proteotyping to Establish Gene Origin within Reassortant Influenza Viruses
Source: PLoS One. 2011 Jan 31;6(1):e15771. doi: 10.1371/journal.pone.0015771 (PMC3031537; doi:10.1371/journal.pone.0015771)
Supplement: Table S2 — Matrix M1 protein derived peptides from the tryptic digestion of the M1 band of the NYMC X-181 reassortant strain as detected by MALDI-MS. (DOC) [file pone.0015771.s002.doc]

Table S2 Matrix M1 protein derived peptides from the tryptic digestion of the M1 band of the NYMC X-181 reassortant strain as detected by MALDI-MS

| ***m/z* [M+H]+**  **monoisotopic**  **(experimental)** | **Δ (ppm)** | **residues** | **missed**  **cleavage**  **sites** | **amino acid sequence** | **modifications*** |
| --- | --- | --- | --- | --- | --- |
| 544.3953 | 4.32 | 102-105 | 2 | KLKR |  |
| 555.2657 | 4.20 | 175-178 | 0 | HENR ** |  |
| 629.3868 | 4.05 | 73-77 | 1 | GLQRR ** |  |
| **749.4692** | **3.26** | **96-101** | **1** | **AVKLYR** |  |
| **849.4093** | **2.71** | **244-250** | **0** | **MGVQMQR **** | **also as Mox** |
| **863.4253** | **3.10** | **211-217** | **0** | **QMVQAMR** | **also as Mox, 2xMox, PyroGlu** |
| 902.4755 | 2.72 | 106-113 | 0 | EITFHGAK ** |  |
| 1005.5110 | 2.88 | 243-250 | 1 | RMGVQMQR | also as 2xMox |
| 1058.5774 | 3.02 | 105-113 | 1 | REITFHGAK |  |
| 1125.7025 | 3.11 | 48-57 | 0 | TRPILSPLTK ** |  |
| 1156.5642 | 3.46 | 244-252 | 1 | MGVQMQRFK | only as Mox |
| 1255.6686 | 3.58 | 218-230 | 0 | TIGTHPSSSAGLK |  |
| **1273.6963** | **2.36** | **164-174** | **0** | **QMVTTTNPLIR** | **also as  Mox, PyroGlu** |
| 1473.7516 | 2.58 | 175-187 | 1 | HENRMVLASTTAK ** | only as Mox |
| **1506.7562** | **3.52** | **36-47** | **0** | **NTDLEVLMEWLK **** | **only as Mox** |
| **1546.8255** | **2.00** | **22-35** | **1** | **AEIAQRLEDVFAGK** |  |
| **1604.8430** | **2.43** | **231-243** | **1** | **NDLLENLQAYQKR** |  |
| 1635.9155 | 3.06 | 58-72 | 0 | GILGFVFTLTVPSER ** |  |
| **1653.8908** | **3.33** | **161-174** | **1** | **SHRQMVTTTNPLIR** | **also as Mox** |
| **1792.9154** | **1.73** | **164-178** | **1** | **QMVTTTNPLIRHENR** | **only as  Mox, PyroGlu** |
| **1863.8369** | **4.24** | **79-95** | **0** | **FVQNALNGNGDPNNMDK **** | **only as Mox** |
| **2019.9362** | **3.02** | **78-95** | **1** | **RFVQNALNGNGDPNNMDK** | **only as Mox** |
| **2276.1087** | **1.63** | **114-134** | **0** | **EISLSYSAGALASCMGLIYNR** | **also as  Mox, PyroGlu** |
| **2383.0385** | **2.60** | **188-210** | **0** | **AMEQMAGSSEQAAEAMEVASQAR** | **also as  Mox, 2xMox, 3xMox** |
| **2685.3940** | **3.65** | **218-242** | **1** | **TIGTHPSSSAGLKNDLLENLQAYQK** |  |
| **2841.4943** | **3.17** | **218-243** | **2** | **TIGTHPSSSAGLKNDLLENLQAYQKR** |  |
| **2851.3269** | **3.40** | **135-160** | **0** | **MGAVTTEVAFGLVCATCEQIADSQHR** | **also as Mox** |
| **3259.4320** | **1.57** | **188-217** | **1** | **AMEQMAGSSEQAAEAMEVASQARQMVQAMR** | **only as  2xMox, 3xMox** |
| **3285.5095** | **-3.74** | **179-210** | **1** | **MVLASTTAKAMEQMAGSSEQAAEAMEVASQAR** | **also as Mox, 2xMox, 3xMox** |

- Modifications Mox, 2xMox, PyroGlu and carbamido denote the oxidationand dioxidation of methionine residues, the presence of a pyroglutamic acid residue, and the carbamidomethyl alkylation of a cysteine residue respectively.

** Denotes a M1 type A signature peptide, or a peptide containing a M1 signature, within 2009 H1N1 pandemic influenza virus (Schwahn et al., 2010a)

Peptides in bold are only found in A/PuertoRico/8/34 originating strain

Peptides in bold with underlined sequences are unique to reassortant strain NYMC X-181
